# Homologous Gold Nanoparticles and Nanoclusters Composites with Enhanced Surface Raman Scattering and Metal Fluorescence for Cancer Imaging

**DOI:** 10.3390/nano8100819

**Published:** 2018-10-11

**Authors:** Xiaoxia Wu, Yan Peng, Xiaomei Duan, Lingyan Yang, Jinze Lan, Fu Wang

**Affiliations:** 1 Laboratory of Environmental Sciences and Technology, Xinjiang Technical Institute of Physics & Chemistry, Chinese Academy of Sciences, Urumqi 830011, China; wuxx@ms.xjb.ac.cn (X.W.); pengyan_15@163.com (Y.P.); duanxiaomei18@mails.ucas.edu.cn (X.D.); yanglingyan16@mails.ucas.edu.cn (L.Y.); lanjinze16@mails.ucas.edu.cn (J.L.); 2 University of Chinese Academy of Sciences, No. 19 Yuquan Road, Beijing 100049, China

**Keywords:** surface-enhanced Raman scattering (SERS), metal-enhanced fluorescence (MEF), dual functional imaging nanoprobe

## Abstract

A large number of deaths from cancer can be attributed to the lack of effective early-stage diagnostic techniques. Thus, accurate and effective early diagnosis is a major research goal worldwide. With the unique phenomenon of localized surface plasmon resonance (LSPR), plasmonic nanomaterials have attracted considerable attention for applications in surface-enhanced Raman scattering (SERS) and metal-enhanced fluorescence (MEF). Both SERS and MEF are ultra-sensitive methods for the detection and identification of early tumor at molecular level. To combine the merits of the fast and accurate imaging of MEF and the stable and clear imaging of SERS, we propose a novel dual functional imaging nanoprobe based on gold nanoparticles and gold nanocluster composites (denoted AuNPC-RGD). The gold nanoparticles are used as LSPR substrates to realized enhancement of Raman or fluorescence signal, while the gold nanoclusters serve as a fluorophore for MEF imaging, and exhibit better biocompatibility and stability. Furthermore, target molecule of cyclic Arg-Gly-Asp (cRGD) is incorporated into the composite to improve delivery efficiency, selectivity and imaging accuracy. These integrated properties endow AuNPC-RGD composites with outstanding biocompatibility and excellent imaging abilities, which could be used to achieve accurate and effective diagnosis for early cancer.

## 1. Introduction

Cancer has become one of the most serious causes of disease-related death, accounting for about 15% of total human deaths every year [1,2,3,4]. The earlier cancer is discovered, the greater the likelihood of successful treatment. Therefore, effective diagnosis for early cancer is of great importance [5]. Despite recent advances in traditional clinical diagnostic techniques (including magnetic resonance imaging, ultrasound imaging, computed tomography and positron emission tomography), the accuracy and sensitivity of diagnosis are still poor during the early stages of cancer, when the tumor is only a few cells in size [6,7]. In this context, the combination of enhanced Raman scattering and fluorescence methods, which uses nanomaterials as a probe for optical image of early-stage cancer, has come to be regarded as a promising alternative strategy, as it can achieve single-molecule imaging with excellent sensitivity and selectivity [8,9,10,11,12].

With the unique phenomenon of localized surface plasmon resonance (LSPR), plasmonic nanoparticles are widely used as a probe in surface-enhanced Raman scattering (SERS) and metal-enhanced fluorescence (MEF) techniques [13,14,15]. SERS can provide ultra-sensitive characterization down to the single-molecular level, and a higher sensitivity (10^10^–10^14^ times enhancement) compared to conventional Raman spectroscopy [16,17,18]. Jing et al. demonstrated the ability of a nanothermometer, which used a gold nanostar-indocyanine nanoprobe to realize real-time monitoring via SERS imaging [19]. However, the drawback of traditional SERS imaging is the long time required for image acquisition. MEF is capable of fluorescence enhancement via the interactions of fluorophores with metallic nanoparticles, and has attracted widespread interest as a method for developing novel nanostructures for biosensors and biomedical engineering [20,21,22]. Lee reported a fast and facile MEF optical method to monitor and probe bacterial interactions in three-dimensional resolution [23]. But the organic fluorophores are relatively unstable against photobleaching and can be easily degraded in microenvironments. Therefore, to achieve both more stable and faster imaging, one highly effective strategy is to combine SERS and MEF to construct a dual functional probe, which would not only obtain ultrahigh-resolution imaging in a short time, but also maintain image stability in the long term.

Herein, we designed and synthesized a novel dual functional nanoprobe combining SERS and MEF for the accurate imaging of early cancer or metastasis tissues. Specifically, gold nanoparticles (AuNPs), used as the source of plasmonic resonance, were modified with a Raman reporter molecule 4-mercaptobenzoic acid (AuNP-MBA), and encapsulated with silica (AuNP@SiO_2_). Then, gold nanoclusters (AuNCs) were grown on surface of AuNP@SiO_2_ (AuNPC), and functionalized with bovine serum albumin (BSA) and cyclic Arg-Gly-Asp (cRGD) to construct the final dual functional nanoprobes (AuNPC-RGD). The novel nanoprobe design combines four advantages. Firstly, the AuNPs used as plasmonic substrates to enhance the signal intensity of Raman activity or fluorescence and the AuNCs which act as a fluorophore for MEF imaging, are homologous nanomaterials possessing good biocompatibility. Secondly, compared with conventional organic dyes, AuNCs exhibit much higher stability, which improves the temporal resolution of imaging. Thirdly, the nanoprobes realize both faster and more accurate MEF imaging in the short term and more stable and clearer SERS imaging in the long term. Finally, the conjugation of the probe with the target molecule, cRGD, improves the delivery efficiency of the latter and therefore the selectivity and imaging accuracy. With these integrated properties, AuNPC-RGD is a novel dual functional imaging agent with outstanding stability and biocompatibility, and excellent SERS and MEF imaging efficiency, capabilities that are urgently needed for early cancer diagnosis and imaging.

## 2. Materials and Methods

### 2.1. Materials

Gold (III) chloride trihydrate (HAuCl_4_·3H_2_O), γ-mercaptopropyltrimethoxysilane (MPTES) and sodium citrate dihydrate (Na_3_Ct·2H_2_O) were purchased from Adamas Beta (Shanghai, China). Ammonium hydroxide (NH_3_·H_2_O, 25%), sodium hydroxide (NaOH) and 2-propanol were obtained from Tianjin Chemical Regent Co., Ltd. (Tianjin, China). Tetraethylorthosilicate (TEOS), bovine serum albumin (BSA) and 4-mercaptobenzoic acid (4-MBA) were purchased from Sigma-Aldrich (St. Louis, MO, USA). *N*-hydroxysuccinimide (NHS) and 1-ethyl-3-(3-dimethylaminopropyl) carbodiimide hydrochloride (EDC·HCl) were obtained from J&K Scientific Ltd. (Beijing, China). Cyclic Arg-Gly-Asp (cRGD) was obtained from GL Biochem Ltd. (Shanghai, China).

### 2.2. Synthesis of the AuNP-MBA

The AuNP-MBA was synthesized by two steps. First, HAuCl_4_ was reduced by Na_3_Ct to form AuNPs [24], 2 mL of HAuCl_4_ solution (10.0 mM) was added into 60 mL of water containing 1 mL of Na_3_Ct (1.0%) and the mixture was heated to boiling for 3.0 min. The color of solution changed to red and the resulted AuNPs dispersion were kept in 4 °C after cooled down to room temperature. Second, the modification of 4-MBA was completed according to a reported approach [25]. 20 µL of different concentration of 4-MBA (1.0–100 mM in ethanol) was mixed with AuNPs dispersion (2 mL) and reacted for 2 min. The SERS intensity of obtained AuNP-MBA1-4 were taken by Raman spectrometer.

### 2.3. Preparation of the AuNP@SiO_2_ and AuNPC

AuNP@SiO_2_ was prepared using TEOS as silicon source with a modified approach [26]. Briefly, 6 mL of AuNP-MBA and 0.75 mL of NH_3_·H_2_O (25%) were added into 30 mL of 2-propanol and then 10% TEOS in ethanol (21–36 μL) was added drop by drop. After the reaction of 5 h, the obtained AuNP@SiO_2_ was washed twice with ethanol by centrifugation (11000 rpm, 10 min) and dispersed in 3 mL of ethanol. For mercapto modification, 3 mL of the above-prepared AuNP@SiO_2_ was mixed with 1 mL of MilliQ water and 30 μL of MPTES. The mixture was kept stirring at 60 °C for 5.0 h in a constant temperature bath and the obtained nanoparticles were collected by centrifugation (11,000 rpm, 10 min) and washed with water for three times, and finally re-dispersed into 3 mL of water.

AuNPC was prepared according to a modified method [27]. 2 mL of the as-prepared mercapto functionalized AuNP@SiO_2_ (AuNP@SiO_2_-SH) and 0.5 mL of HAuCl_4_ (10.0 mM) were mixed for 30 min, and 0.5 mL of BSA (50 mg/mL) was added into the solution. After 10 min, 0.2 mL of NaOH aqueous solution (1 M) was injected and the mixture was kept at 37 °C for 3 h. Subsequently, the resulting AuNPC was collected by centrifugation (12,000 rpm for 10 min) and re-dispersed into 2 mL of water. Then, the MEF intensity of AuNPC1-4 were taken by fluorescence spectrometer.

### 2.4. Conjugation of Targeted Molecule cRGD

Finally, the target molecule cRGD was grafted to the AuNPC nanoparticles with a modified approach [28,29]. Specifically, 2 mL of as-prepared AuNPC, 4 mg of EDC·HCl and 2.5 mg of NHS were mixed in 8 mL of PBS (pH 7.4) for the reaction of 8 h. Then, 2 mg of cRGD was added into the solution and kept stirring in the dark at room temperature. After 16 h, the resulted AuNPC-RGD was collected by centrifugal filter units (Millipore, MWCO 50.0 kDa, Darmstadt, Germany) and dispersed in 2.0 mL of water for subsequent application.

### 2.5. Characterization

Transmission electron microscope (TEM) images of the nanoparticles were obtained by a Tecnai G20 (FEI, Hillsboro, OR, USA), which was operated at 200 kV. The size distribution and zeta potential were measured by dynamic light scattering (DLS) with a zeta particle size analyzer (Nano-ZS, Malvern, UK). Raman spectra and imaging were taken with a confocal microprobe Raman system (LabRAM HR Evolution, Tokyo, Japan) with 633 nm laser, and scattering spectra were recorded in the range of 400–1700 cm^−1^. The characterization of fluorescence spectra were taken on fluorescence spectrophotometer (F-7000, Hitachi, Tokyo, Japan), and fluorescence imaging was taken with a confocal laser scanning microscope (CLSM, Nikon C2 SIM, Tokyo, Japan).

### 2.6. Biocompatibility and Cellular Uptake of Nanoparticles

Human cervical cancer cell line HeLa cells were cultured in DMEM at 37 °C in 5% CO_2_.

For the evaluation of in vitro cytotoxicity, the MTT assay was performed on the HeLa cells [3]. Briefly, 100 μL of HeLa cells (1 × 10^5^ cells per mL) was added in 96-well plate with the incubation of 24 h. Then the culture medium was removed and 100 μL of AuNPC-RGD (0–1.0 mg/mL) in fresh culture medium was added and incubated for another 24 h. Next 10 μL of MTT (5 mg/mL) was added and incubated for 4 h, then 100 μL of DMSO was added after removing the medium and free MTT. The absorbance of the suspension was recorded by a microplate reader (Thermo MultiskanFC, Waltham, MA, USA) at a wavelength of 570 nm.

To measure the cellular uptake of nanoparticles, inductively coupled plasma (ICP) analysis was used. HeLa cells were incubated with either AuNPC or AuNPC-RGD (5 mg/mL) as described above for 2 or 4 h. Then HeLa cells were washed for three times by PBS and collected by centrifugation (1000 rpm, 5 min). Finally, the amount of Au of cell samples were measured by ICP-OES (VISTA-PRO CCD Simultaneous ICP-OES, VARIAN, Palo Alto, CA, USA).

### 2.7. Surface-Enhanced Raman Scattering (SERS) and Metal-Enhanced Fluorescence (MEF) Imaging

For the study of SERS imaging, 2 × 10^5^ HeLa cells dispersed in 2 mL of culture medium were added in a 6-well plate and incubated for 24 h. Then, the culture medium was replaced with 2 mL of fresh culture containing AuNPC or AuNPC-RGD (5 mg/mL). After another 4 h, HeLa cells were washed for three times with PBS. SERS images at the Raman shift of 1065–1080 cm^−1^ were then obtained under laser excitation at 633 nm on the confocal laser Raman scanning microscopy (LabRAM HR Evolution, Tokyo, Japan).

A similar method was used to study the MEF imaging. Additionally, the HeLa cells were treated with Hoechst 33258 (0.5 μg/mL) to stain the nucleus. Hoechst and AuNPC were excited at the same wavelength of 405 nm, and the fluorescence images were taken by a CLSM (Nikon C2 SIM, Tokyo, Japan) at wavelengths of 420–480 and 600–660 nm.

## 3. Results and Discussion

### 3.1. Characterization of the Dual Functional Nanoprobes

**Scheme 1:**
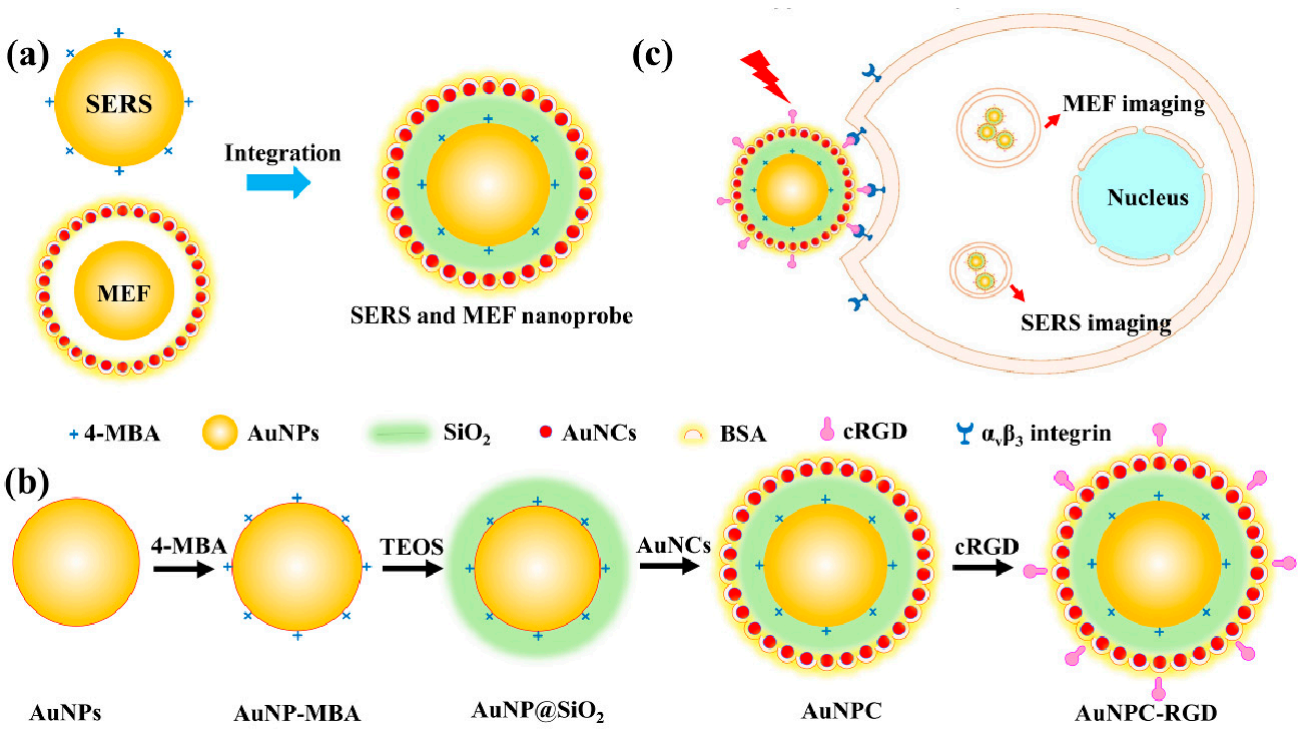
The illustration for the design (**a**), preparation (**b**) and application (**c**) of the dual-functional imaging nanoprobe.

The design, preparation and application of the SERS nanoparticles are illustrated in Scheme 1. As shown in Scheme 1a, the AuNPs are used as LSPR substrates to enhance the signal intensity of Raman scattering and fluorescence. In combination with the enhanced Raman and fluorescence signals, the novel dual functional nanoprobe is designed to simultaneously achieve SERS and MEF imaging. As shown in Scheme 1b, the LSPR substrate of AuNPs are modified with a Raman reporter molecule of 4-MBA, via an Au-S bond to realize SERS imaging. To avoid fluorescence quenching by the AuNPs, the surfaces of AuNP-MBA are encapsulated in SiO_2_ shells, for which the thickness of SiO_2_ is optimized with respect to fluorescence enhancement. Then, the AuNP@SiO_2_ are functionalized with the –SH group to induce the growth of AuNCs on the nanoparticles. By S–Au bonds, both BSA (containing S–S) and AuNP@SiO_2_ (containing –SH) could form S–Au bonds with AuNCs, which modified BSA on the surface of AuNP@SiO_2_ via AuNCs. The BSA of AuNCs are attached on the surface of the dual-functional nanoparticles to improve the stability and biocompatibility of the nanoparticles and achieve MEF imaging. Then, the target molecule, cRGD, which has been demonstrated to show high binding affinity to α_v_β_3_ integrin that are up-regulated by tumor endothelial cells, is conjugated onto the surface of the AuNPC through the amidation reaction between –COOH groups of BSA and –NH_2_ groups of cRGD [29]. Scheme 1c shows the application of AuNPC-RGD for targeted imaging of cancer cells, which achieve excellent SERS and MEF imaging ability and efficiency.

**Figure 1:**
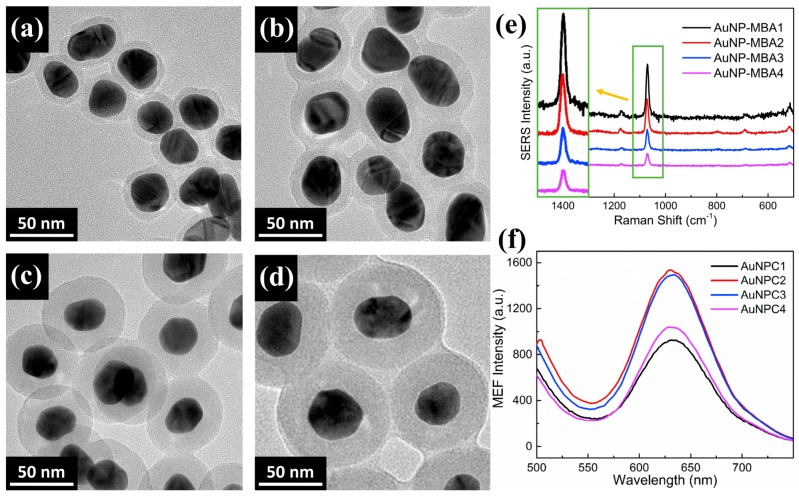
(**a**–**d**) TEM imaging of AuNP@SiO_2_-1-4; (**e**) SERS spectra of the AuNP-MBA1-4; (**f**) MEF spectra of the AuNPC1-4.

The amount of 4-MBA on the AuNPs is a very important parameter for the SERS effects of LSPR system, so that the concentration of 4-MBA during the preparation of AuNP-MBA nanoparticles were systematically optimized with respect to the stability and SERS intensity of the AuNP-MBA nanoparticles. In Table 1 and Figure 1e, the SERS intensity and spectra of preparative conditions and performances of the AuNP-MBA are presented. The major Raman peaks of AuNP-MBA1-4 at 1076 cm^−1^ are magnified and shown in Figure 1e which indicate the vibration peak of benzene ring of 4-MBA [25]. It could be found that the tendency of SERS intensity increased obviously with the increase of 4-MBA concentration from 10 to 500 µM (the magnified spectra in Figure 1e & Table 1) for AuNP-MBA1-4. On the basis of the stability and SERS intensity of the modified AuNP-MBA nanoparticles, the final concentration of 4-MBA was fixed at 500 μM in all subsequent experiments, due to the higher 4-MBA concentrations resulted in poor stability of the nanoparticles (the AuNPs was easy to agglomerate with 4-MBA at concentration of 1 mM so that its SERS intensity was not measured). To prevent fluorescence quenching, the surfaces of the AuNP-MBA (about 35 nm) were encapsulated with the SiO_2_ shells. The thickness of the SiO_2_ shells was optimized with respect to the MEF intensity of the nanoparticles by varying the concentration of TEOS, as shown in the TEM images of Figure 1a–d. Table 2 shows the performances of the AuNPC nanoparticles and Figure 1f shows the MEF spectra of the prepared AuNPC nanoparticles. With the increase of SiO_2_ shell thickness (from about 6.3 to 20 nm, as shown in Table 2), the MEF intensities of the AuNPC nanoparticles first increased and then decreased. And the peak value of MEF were shown in Table 2, AuNPC2 and AuNPC3 had similar MEF intensity, but AuNPC2 had higher MEF and the thickness of SiO_2_ in AuNPC2 was optimization condition for MEF. Therefore, SiO_2_ shells of thickness (13 ± 1.2 nm) were chosen to maximize the fluorescence enhancement for the following application of MEF imaging.

**Figure 2:**
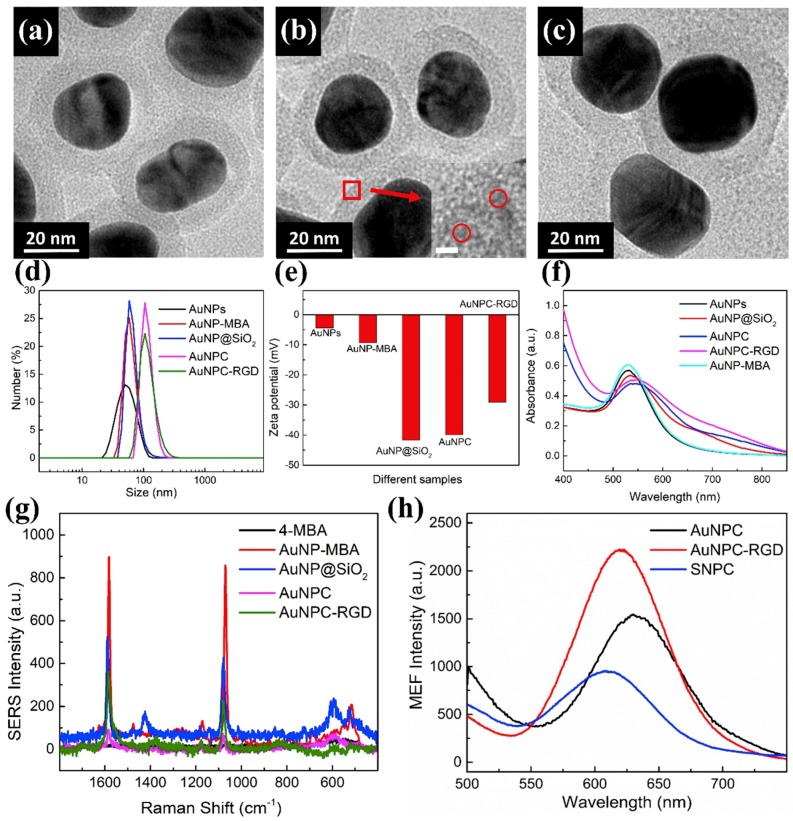
Characterization of the bifunctional nanoprobes. (**a**–**c**) TEM images of the AuNP@SiO_2_, AuNPC and AuNPC-RGD; (**d**–**e**) Size distributions and zeta potentials of the AuNPs, AuNP-MBA, AuNP@SiO_2_, AuNPC and AuNPC-RGD in water; (**f**) UV-Vis spectra of the AuNPs, AuNP-MBA, AuNP@SiO_2_, AuNPC and AuNPC-RGD in aqueous solution; (**g**) SERS spectra of the 4-MBA, AuNPs, AuNP@SiO_2_, AuNPC and AuNPC-RGD; (**h**) MEF spectra of the AuNPC, AuNPC-RGD and SNPC in aqueous solution.

Figure 2a–c show TEM images of the modified AuNPs, exhibiting their core-shell structure. It can be seen that the AuNP@SiO_2_, AuNPC and AuNPC-RGD are all well dispersed. The SiO_2_ was modified with –SH groups which could link AuNCs by S–Au bonds and anchored in the surface of SiO_2_ shell. The size of AuNCs was below 2 nm which was too small to be seen [27]. When it was enlarged (the insert image of Figure 2b), AuNCs could be found on the SiO_2_ shell in AuNPC nanoparticles, indicating that the AuNCs were modified on AuNPC successfully. Figure 2d shows the size distributions of the AuNPs, AuNP-MBA, AuNP@SiO_2_, AuNPC and AuNPC-RGD in MilliQ water. The hydrodynamic diameters of the nanoparticles increase in the order AuNPs < AuNP-MBA < AuNP@SiO_2_ < AuNPC < AuNPC-RGD, i.e., the nanoparticles have successively larger diameters after each modification step. Both the TEM and DLS results indicate that the size of the AuNPC-RGD nanoparticles is less than 200 nm, which is sufficiently small to allow enhanced permeability and retention effect for targeting tumor cells [7]. As shown in Figure 2e, the zeta potentials of the AuNPs, AuNP-MBA, AuNP@SiO_2_, AuNPC and AuNPC-RGD are −4.2 mV, 9.7 mV, −42.3 mV, −41.5 mV and −29.8 mV, respectively. All of the nanoparticles are therefore negatively charged, which can be expected to suppress their non-specific interaction with cells [24,25]. Figure 2f shows the UV-Vis spectra of the AuNPs, AuNP-MBA, AuNP@SiO_2_, AuNPC and AuNPC-RGD. It could be found that the absorbance peak of AuNPs at 525 nm with the wavelength range of 460–700 nm, indicating that AuNPs had a strong plasmonic absorption peak in LSPR [14]. And strong resonance effect was induced with the incident light of 525 nm which could produce high SERS effect [17]. In consideration of fluorescence interference of cells, long wavelength of 633 nm laser was used to excite SERS imaging. The absorption peak shifts from 525 nm for the AuNPs to 550 nm for the AuNPC-RGD. This red shift of surface plasmon band can be ascribed to modification of 4-MBA, SiO_2_ shell, AuNCs and cRGD which changed the refractive index surrounding the AuNPs [30]. The modification of 4-MBA on AuNPs does not change the plasmonic absorption of AuNPs due to the low concentration of 4-MBA encoded on AuNPs. When encapsulated with AuNCs and cRGD, the absorption at range of long wavelength (650–800 nm) increased obviously, which resulted from the interaction between the LSPR effects of AuNPs and AuNCs [31]. Figure 2g shows SERS spectra of 4-MBA, AuNP-MBA, AuNP@SiO_2_, AuNPC and AuNPC-RGD. The peaks of 1076 cm^−1^ associated with vibration peak of benzene ring, and 1581 cm^−1^ were identified along with the band at 1422 cm^−1^ corresponding to deformation and stretching vibrations of carboxylate groups [32]. It is clear that Raman intensity of 4-MBA increases due to the SERS of AuNPs, and the enhancement factor (EF) of SERS for AuNPC-RGD is calculated according to the equation (EF = [*I*_SERS_]/[*I*_Raman_] × [*N*_Raman_]/[*N*_SERS_]) [18,33]. The Raman intensity of 4-MBA or AuNPC-RGD are *I*_Raman_ or *I*_SERS_, and *N*_Raman_ or *N*_SERS_ indicated molecular weight of 4-MBA in dispersion or on AuNPC-RGD. The EF is calculated to be 500, suggesting that the intensity of AuNP-MBA is 500 times greater than that of 4-MBA without AuNPs substrates. Figure 2h exhibits MEF spectra of AuNPC, AuNPC-RGD and silica nanospheres (~70 nm) without AuNPs core that grow gold nanoclusters on the surface (abbreviated as SNPC). The fluorescence property of AuNCs mostly relate to ligands with Au atoms, which influence the emission wavelength and intensity of AuNCs [21]. The fluorescence peak of AuNCs shifted when modified cRGD, it could be ascribed to the ligand molecule of BSA changed to BSA-cRGD which had great influence on luminous properties of AuNCs. Moreover, the AuNPs could also influence the emission property of AuNCs induced the difference of AuNPC and SNPC. AuNPC-RGD had higher MEF intensity due to the enrichment of AuNPC-RGD after centrifugation. The silica nanoparticles with similar size of AuNP@SiO_2_ (about 70 nm), was prepared by hydrolysis of tetraethylorthosilicate (TEOS) [26]. SNPC (silica nanoparticles modified with AuNCs with the same preparation method with AuNPC) was used as control to calculate EF of MEF for AuNPC. There was no AuNPs core in silica nanoparticles but AuNCs were conjugated on their surfaces. Comparing the fluorescence spectra of AuNPC and SNPC, we can find that the fluorescence intensity is approximately doubled than AuNPC.

### 3.2. Biocompatibility and Cellular Uptake of Nanoparticles

It is well known that biocompatibility is an important factor for any nanoplatforms in a drug delivery system, as it determines whether a nanomaterial can be used in vivo [34]. AuNPC and AuNPC-RGD were modified with BSA, which not only increases the stability and biocompatibility of the nanomaterials, but also provides a large number of functional groups of –COOH and –NH_2_. Through the amidation reaction between –COOH and –NH_2_, the target molecule cRGD was grafted onto the nanoparticles. In preparation of AuNPC-RGD, a tiny amount of cRGD reacted with a high amount of BSA, suggesting most of cRGD reacted with BSA and conjugated on AuNPC. The amount of AuNCs and BSA were all the same, and then cRGD was modified on AuNPC, which could not interfere the amount of cRGD on AuNPC-RGD. cRGD is a typical target molecule possessing high binding affinity to α_v_β_3_ integrin, which are highly expressed in tumor endothelial cells (such as lung adenocarcinoma, cervical cancer and breast cancer cells) [29].

**Figure 3:**
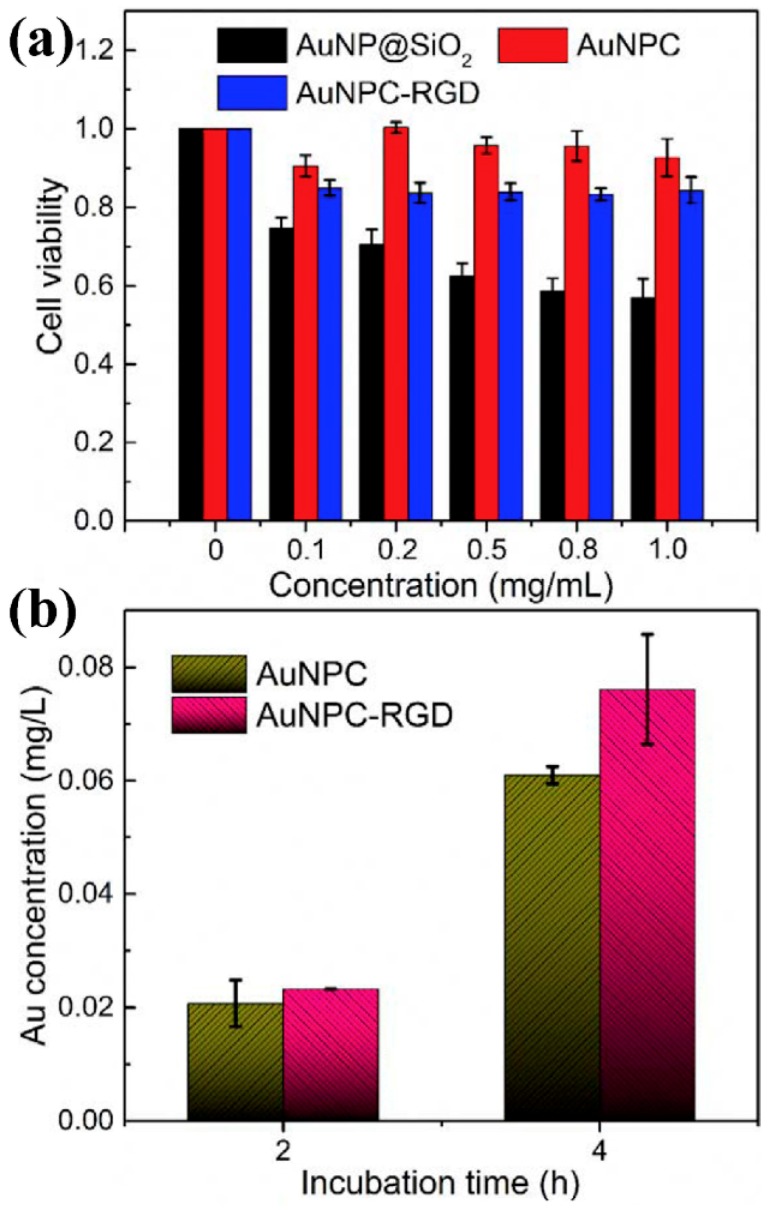
In vitro cytotoxicity and cellular uptake of different nanoparticles. (**a**) Viabilities of HeLa cells incubated with different nanoparticles; (**b**) Cellular uptake performance of AuNPC and AuNPC-RGD with incubation for 2 and 4 h.

The in vitro cytotoxicity and cellular uptake of nanoparticles must be evaluated before their application to ensure that the biocompatibility of nanoparticles could be applied for cell imaging [24]. Firstly, the cytotoxicity of the as-prepared different nanoparticles were evaluated via MTT assay, in which HeLa cells were separately incubated with AuNPs@SiO_2_, AuNPC and AuNPC-RGD. From Figure 3a, it can be observed that AuNPs@SiO_2_ had the most severe cytotoxicity (survival rates of 74.7% for 0.1 mg/mL and 57.1% for 1 mg/mL), while the other nanoparticles had excellent biocompatibility (survival rates higher than 85.0% for 0.1 mg/mL and 83.3% for 0.8 mg/mL for both materials). Nonetheless, the cell viability in the presence of AuNPC-RGD, in which cRGD was conjugated onto the nanoparticles, was reduced (compared with 92.65% for 1 mg/mL of AuNPC), because the cellular uptake of the former was higher than the latter, as shown in Figure 3b. Figure 3b shows the quantitative ICP measurement of AuNPC and AuNPC-RGD accumulated in cells. The cellular uptake of the nanoparticles increased with the increase of incubation time from 2 h to 4 h. And the cellular uptake of AuNPC-RGD increased compared with AuNPC, which indicates that it could realize active targeting for AuNPC-RGD delivery to tumor cells.

### 3.3. Application of SERS and MEF Imaging

As is widely known, AuNPs have strong plasmonic properties through their long electronic relaxation time, which provide powerful enhancement for Raman scattering and fluorescence signals [18]. As the SERS and MEF techniques possess ultrahigh-sensitivity that can reach single molecule detection, the prepared AuNPC-RGD has the potential to be used in single cell imaging.

**Figure 4:**
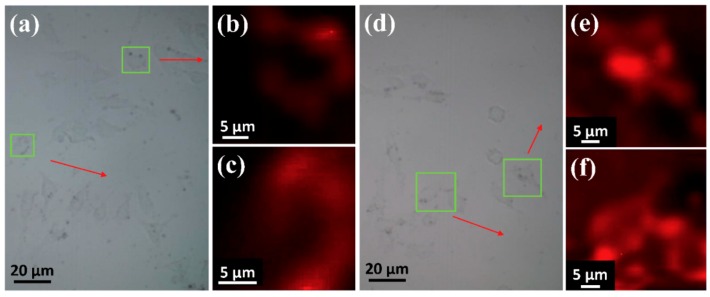
SERS images of HeLa cells incubated with nanoprobes. (**a**–**c**) AuNPC; (**d**–**f**) AuNPC-RGD. SERS images at the Raman shift of 1065–1080 cm^−1^ were obtained under the laser of 633 nm.

To investigate the performance of the nanoprobe for SERS imaging, HeLa cells were incubated with AuNPC or AuNPC-RGD. Figure 4a shows the bright field cell imaging of HeLa cells incubated with AuNPC, and Figure 4b,c are the corresponding SERS images, in which the Raman signals were collected in the range of 1065–1080 cm^−1^ and the excitation wavelength was chosen as 633 nm to avoid fluorescence interference [19]. The SERS images of Figure 4b,c are enlarged views of the cells in the green boxes in Figure 4a, which are well matched with the corresponding bright field images. SERS images of HeLa cells incubated with AuNPC-RGD are exhibited in Figure 4d–f. Similar to AuNPC, Figure 4d shows the bright field imaging of the cells and Figure 4e,f are the SERS images under the same conditions as mentioned above, and both the bright field and SERS images are again completely matched. Compared with Figure 4b,c, the SERS intensities of Figure 4e,f are much brighter, which can be ascribed to the higher cellular uptake of AuNPC-RGD than AuNPC. The very close matching of the SERS images with the bright field images indicates that the SERS nanoprobes can achieve single-cell imaging.

**Figure 5:**
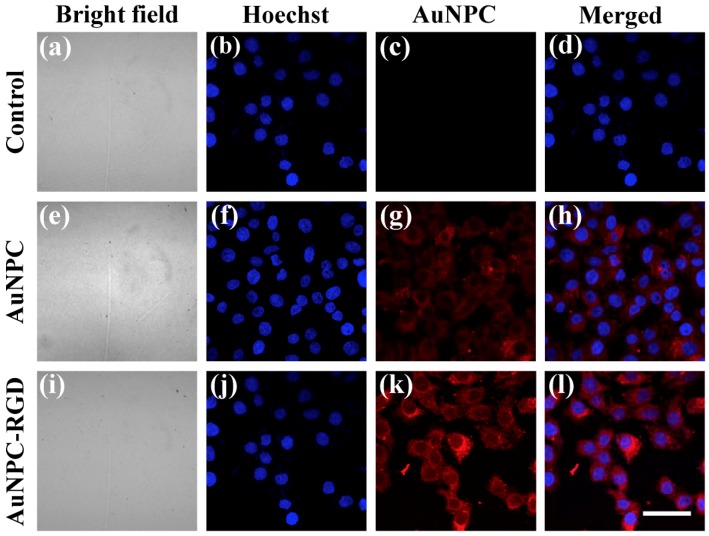
Laser confocal microscopy images of HeLa cells incubated with/without nanoparticles. (**a**–**d**) Without nanoparticles; (**e**–**h**) with AuNPC; (**i**–**l**) with AuNPC-RGD (**i**–**l**). Hoechst and AuNPC were excited at the same wavelength of 405 nm, and the fluorescence images were taken at 420–480 and 600–660 nm, respectively. Scale bar: 50 μm.

Compared with Raman scattering signals, fluorescence signals are stronger and more sensitive [35]. By utilizing the MEF effect, fluorescence intensity and photostability can be further increased [18]. As presented in Figure 5, the as-prepared nanoparticles show excellent MEF performance. Figure 5a–d show the HeLa cells incubated without nanoparticles as a control for comparison with AuNPC and AuNPC-RGD. The bright field and MEF images of HeLa cells incubated with AuNPC or AuNPC-RGD are as shown in Figure 5e–l, respectively. The images of bright field were shown clearly in Figure S1–S3 (Supplementary Materials). The images of the second columns (Figure 5b,f,j) and third columns (Figure 5c,g,k) are fluorescence images of Hoechst 33258 (blue) and AuNPC (red) under single-wavelength 405 nm excitation. The MEF image of AuNPC-RGD displays stronger fluorescence than the image of AuNPC, as a result of the active-targeting effect of AuNPC-RGD. From the merged images (Figure 5d,h,l), the cell nucleus (blue) and cytoplasm (red) are matched accurately, indicating that the MEF nanoprobes can achieve single-cell fluorescence imaging. The results of SERS and MEF imaging demonstrate that AuNPC-RGD as a dual-functional imaging agent shows outstanding biocompatibility and excellent SERS and MEF imaging efficiency, and is expected to be applied for effective cancer imaging.

## 4. Conclusions

In summary, we have developed new nanoprobe for SERS and MEF dual functional imaging to achieve fast and long-lasting imaging for early cancer. AuNPs of about 35 nm in size are modified with a Raman reporter molecule of 4-MBA, encapsulated with SiO_2_, grown AuNCs on the surface, and functionalized with BSA and cRGD to construct the dual functional (i.e., SERS and MEF) probes, denoted AuNPC-RGD. To maximize the stability, SERS intensity and MEF intensity of the AuNPC-RGD nanoparticles, the concentration of 4-MBA was optimized to be 500 µM, and the thickness of SiO_2_ was optimized to be about 13 nm. The TEM and DLS results indicate the monodisperse AuNPC-RGD, with a negative surface charge that suppresses the non-specific interaction with cells. From the SERS and MEF spectra, the calculated EFs of AuNPC-RGD are 500 and 2, respectively. Moreover, cell viability in the presence of 1 mg/mL AuNPC-RGD is 84.3%, indicating the outstanding biocompatibility of the nanoprobe. For application in SERS and MEF imaging, our AuNPC-RGD nanoparticles exhibit excellent imaging abilities, which suggest that the AuNPC-RGD composite is a powerful probe for accurate and effective cancer imaging.

## Figures and Tables

**Table 1:** The conditions and results for the preparation of the AuNP-MBA.

Nomenclature	C_Au_ (mg/mL)	C_4-MBA_ (μM)	SERS Intensity ^a^
**AuNP-MBA1**	0.05	500	964 ± 2.5
**AuNP-MBA2**	0.05	100	627 ± 5.2
**AuNP-MBA3**	0.05	50	215 ± 6.7
**AuNP-MBA4**	0.05	10	23 ± 4.3

^a^ SERS intensity of the nanoparticles determined at 1076 cm^−1^.

**Table 2:** The conditions and results for the preparation of the AuNPC.

NO.	Size (nm)	The Thickness of Shell (nm)	MEF Intensity ^a^
**AuNPC1**	45 ± 1.9	6.3 ± 0.8	930 ± 3.2
**AuNPC2**	60 ± 2.3	13 ± 1.2	1545 ± 4.2
**AuNPC3**	70 ± 2.9	17 ± 1.3	1480 ± 4.1
**AuNPC4**	78 ± 4.0	20 ± 2.0	1037 ± 5.0

^a^ MEF intensity of the nanoparticles determined at 630 nm.

## Supplementary Materials

The following are available online at http://www.mdpi.com/2079-4991/8/10/819/s1.
