# Supplementary material for: Homologous Gold Nanoparticles and Nanoclusters Composites with Enhanced Surface Raman Scattering and Metal Fluorescence for Cancer Imaging
Source: Nanomaterials (Basel). 2018 Oct 11;8(10):819. doi: 10.3390/nano8100819 (PMC6215224; doi:10.3390/nano8100819)
Supplement: Supplementary file 1 [file nanomaterials-08-00819-s001.zip › nanomaterials-367640-SI.pdf]

Supporting information

Homologous Gold Nanoparticles and Nanoclusters Composites with Enhanced Surface Raman Scattering and Metal Fluorescence for Cancer Imaging

Xiaoxia Wu [1,2], Yan Peng [1], Xiaomei Duan [1], Lingyan Yang [1], Jinze Lan [1] and Fu Wang [1,*]

[1] Laboratory of Environmental Sciences and Technology, Xinjiang Technical Institute of Physics & Chemistry, Chinese Academy of Sciences, Urumqi, Xinjiang 830011, China; wuxx@ms.xjb.ac.cn (X.W.); yanpeng9236@sina.com (Y.P.); 1498244651@qq.com (X.D.); 994125943@qq.com (L.Y.); 593530519@qq.com (J.L.)
[2] University of Chinese Academy of Sciences, No. 19 Yuquan Road, Beijing 100049, China
* Correspondence: wangfu@sjtu.edu.cn (F.W.); Tel.: +86-021-54742824 (F.W.)

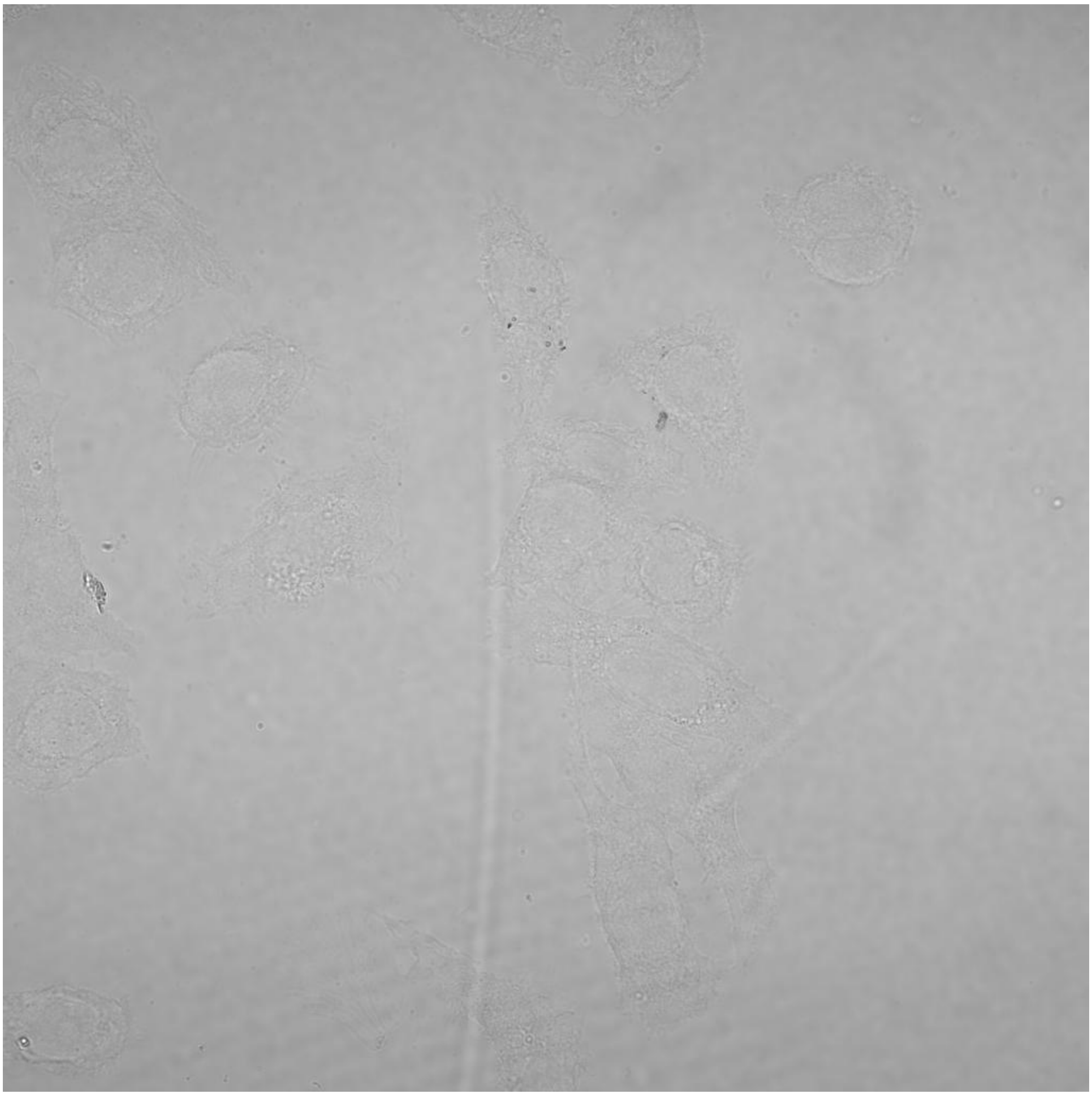

Figure S1. The bright filed of laser confocal microscopy images for HeLa cells incubated without nanoparticles.

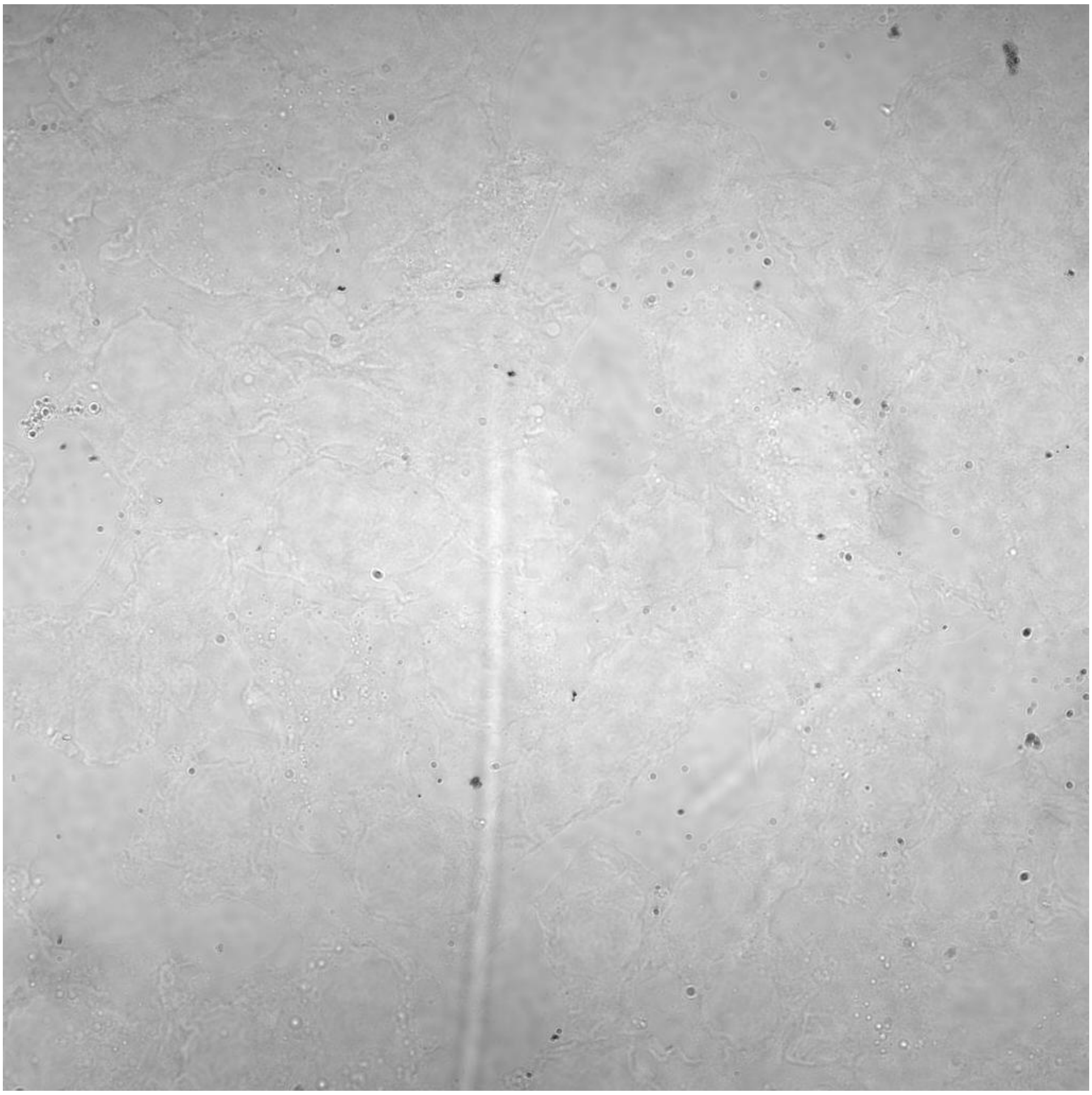

Figure S2. The bright filed of laser confocal microscopy images for HeLa cells incubated with AuNPC nanoparticles.

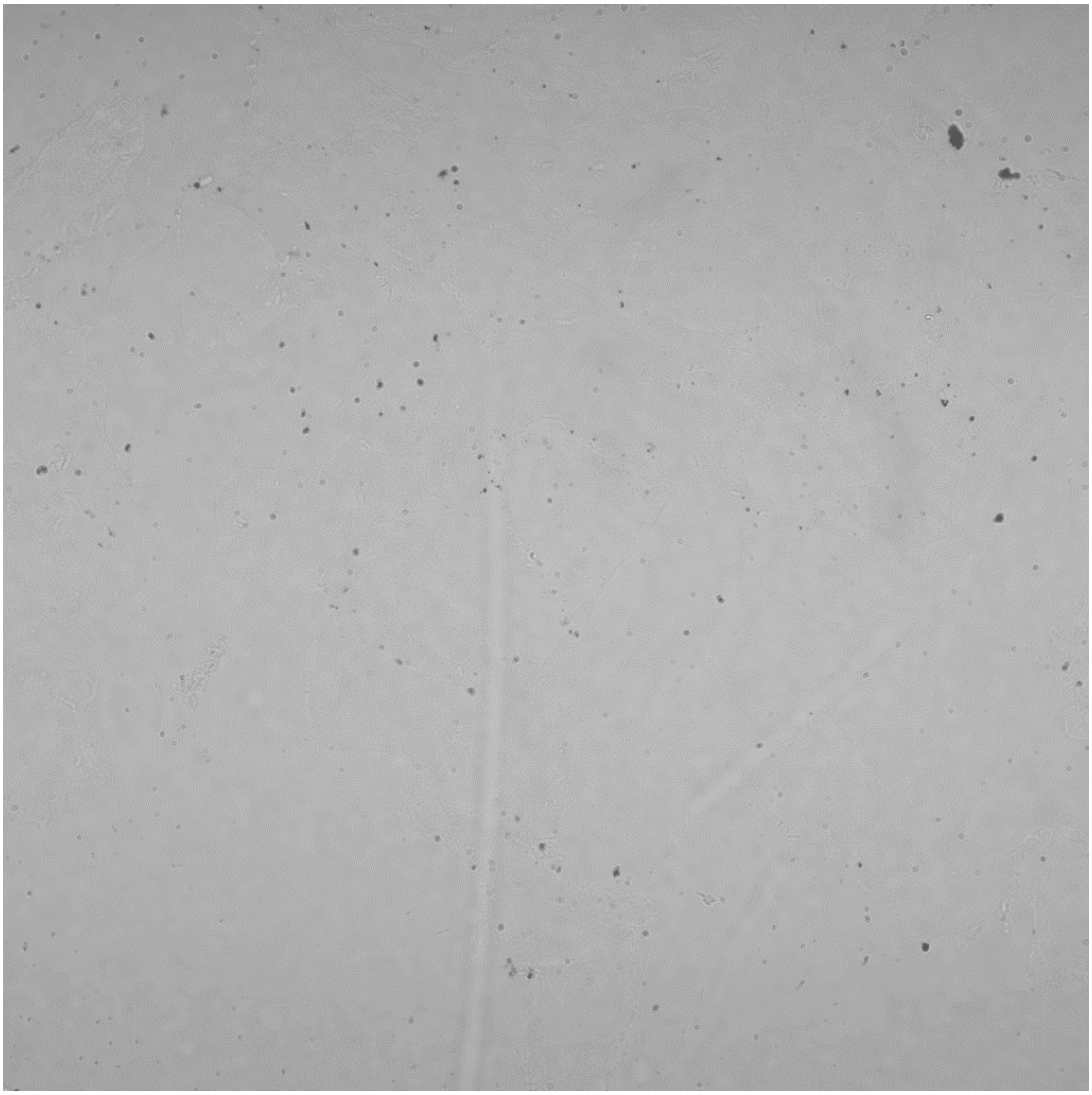

Figure S3. The bright filed of laser confocal microscopy images for HeLa cells incubated with AuNPC-RGD nanoparticles.
